# Machine-learning-based children’s pathological gait classification with low-cost gait-recognition system

**DOI:** 10.1186/s12938-021-00898-0

**Published:** 2021-06-22

**Authors:** Linghui Xu, Jiansong Chen, Fei Wang, Yuting Chen, Wei Yang, Canjun Yang

**Affiliations:** 1 Ningbo Research Institute, Zhejiang University, Ningbo, 315100 China; 2 State Key Laboratory of Fluid Power and Mechatronic Systems, Zhejiang University, Hangzhou, 310027 China; 3 Department of Orthopedics, The Children’s Hospital, Zhejiang University School of Medicine, Hangzhou, 310006 China; 4 Industrial Design Department of the Art and Design Institute, China Academy of Art, Hangzhou, 310024 China; 5 Hebei Heavy Machinery Fluid Power Transmission and Control Lab, Yanshan University, Qinhuangdao, 066004 China

**Keywords:** Pathological gait recognition, Pressure-sensor array, Gait classification, Feature extraction

## Abstract

**Background:**

Pathological gaits of children may lead to terrible diseases, such as osteoarthritis or scoliosis. By monitoring the gait pattern of a child, proper therapeutic measures can be recommended to avoid the terrible consequence. However, low-cost systems for pathological gait recognition of children automatically have not been on market yet. Our goal was to design a low-cost gait-recognition system for children with only pressure information.

**Methods:**

In this study, we design a pathological gait-recognition system (PGRS) with an 8 × 8 pressure-sensor array. An intelligent gait-recognition method (IGRM) based on machine learning and pure plantar pressure information is also proposed in static and dynamic sections to realize high accuracy and good real-time performance. To verifying the recognition effect, a total of 17 children were recruited in the experiments wearing PGRS to recognize three pathological gaits (toe-in, toe-out, and flat) and normal gait. Children are asked to walk naturally on level ground in the dynamic section or stand naturally and comfortably in the static section. The evaluation of the performance of recognition results included stratified tenfold cross-validation with recall, precision, and a time cost as metrics.

**Results:**

The experimental results show that all of the IGRMs have been identified with a practically applicable degree of average accuracy either in the dynamic or static section. Experimental results indicate that the IGRM has 92.41% and 97.79% intra-subject recognition accuracy, and 85.78% and 78.81% inter-subject recognition accuracy, respectively, in the static and dynamic sections. And we find methods in the static section have less recognition accuracy due to the unnatural gesture of children when standing.

**Conclusions:**

In this study, a low-cost PGRS has been verified and realize feasibility, highly average precision, and good real-time performance of gait recognition. The experimental results reveal the potential for the computer supervision of non-pathological and pathological gaits in the plantar-pressure patterns of children and for providing feedback in the application of gait-abnormality rectification.

## Background

Children are prone to have pathological gaits when starting toddling, which may cause osteoarthritis, scoliosis, or other debilitating diseases. To monitoring the pathological gait pattern of a human, various bio-signals are adopted among which kinematics information and plantar-pressure show more potential for their easy to measure and explain [1, 2]. These high-dimensional bio-signals indicate complex states of human muscles and joints [3], which cause difficulty to interpret directly by conventional kinematics or kinetics. However, machine learning (ML) recently has more potential to deal with the large-data-driven pattern-recognition problems with the development of computer technology [1, 4].

Employing computer-vision technology, kinematics information can be easily acquired. Elham et al. acquired gait features including angles, velocity, and acceleration of the joints based on Kinect skeletal tracking sequences [5]. Two ML approaches, an instance-based discriminative classifier and a dynamical generative classifier, were examined to distinguish between healthy and pathological gaits. F1-score of the former can reach up to 96% when walking at a fast pace. Javier et al. developed vision-based gait-impairment analysis for aided diagnosis [6]. A number of semantic and normalized gait features were computed from a single video to provide samples under eight different walking styles: one normal and seven impaired patterns. Several statistical studies were carried out to prove the sensitivity of features in measuring the expected pathologies. Zakaria et al. [7] classified Autism Spectrum Disorder (ASD) children’s gait from normal gait. Gait features were the absolute or relative Cartesian coordinates of 20 joints of the subject measured by a depth camera. Various methods were used to classify these two gaits, among which support vector machine (SVM) classifier has the lowest accuracy, 98.67%, and Naives Bayes classifier has the highest accuracy, 99.66%. Chen et al. [8] proposed a gait classification and develop a simple and efficient method for the quantification method for parkinsonian gait from monocular video imaging based on kernel-based principal component analysis. Faragó et al. [9, 10] proposed a framework for classifying normal walking, heel-walking, and toe-walking based on the cross-correlation of plantar pressures with corresponding lower-limb EMG signals.

Plantar pressure contains abundant gait-pattern information, which can be used to reasonably predict and explain human physiological diseases [2]. However, it has high dimensionality, temporal dependence, high variability, complex correlations between curves, and high non-linear relationship features [4]. Mei et al.[11, 12] analyzed the force of center of pressure (CoP) sample entropy characteristics among the four types, pes cavus, pes valgus, hallux valgus, and normal feet, using the Footscan system. The study showed that dynamic characteristics of CoP progression contain information of the foot type. Zhu et al. [13] developed an umbilical data-acquisition system to measure the pressure between the foot and shoe during walking that had seven force-sensitive resistors (FSRs) on the surface of each insole of a pair of extra-depth shoes. The team found that a shuffling gait with short steps would increase the period of foot flat and thus minimize any excessive local plantar pressures [14]. Lin et al. [15] employed dynamic features derived from tracking gait to recognize individuals. The self-organizing-map (SOM) neural network (NN) algorithm and SVM were used in both schemes for data classification. Experiments showed that a higher recognition rate was achieved with the method using all of the plantar pressure sensor-cell values during walking regardless of the algorithm used, which suggested that the foot-pressure distribution of gait is a suitable feature for gait recognition. Sazonov et al. [16] built a NN to predict geriatric patterns using plantar pressure and heel acceleration information, whose classification accuracy was 91.6% on average. Data for training the NN were collected by sensor shoes with 34 pressure-sensing elements uniformly distributed across the foot and a 2D accelerometer. Based on a shoe-integrated system with an inertial measurement unit (IMU), four FSRs, and a bend sensor, Chen et al. [17] applied principal component analysis (PCA) and SVM for multi-pattern classification (toe-in, toe-out, over-supination, heel-walking, and normal pattern). A total of four subjects tested the shoe-integrated device in outdoor environments. Experimental results of the four subjects demonstrated that the proposed method was robust and highly accurate up to 90%. More interestingly, the study showed that insole sensors played a more important role in solving classification problems than IMUs.

Here, three foot types of children including toe-in, toe-out, and flat feet are concerned. Toe-in, toe-out, and flat feet are the most common reasons for parental concerns and referral for a specialist opinion about their children’s gait [18]. The vast majority of pathological gait may correct spontaneously if left untreated [19–23] due to the underdeveloped skeleton of children, which makes it more of a concern to the parents than to the podiatrist. However, since the theoretical explanation and analysis for the self-healing capability is unclear, the child's gait still needs constant attention. Thus, designing a device for parents to watch children’s gait periodically at home is of high necessity. Many underlying causes leading to in- or out-toeing gait lies in the hip joint, femur, or tibia or the hindfoot or forefoot [18]. The most straightforward physical examination method is measuring foot progression angle (FPA) which describes the orientation of the child’s foot to the direction of progression. For a normal child, FPA is slightly out-toed (+ 10°) with a range from −3° to + 20° [24, 25]. If FPA is less than −3°, a child is considered to have an in-toeing gait and if it is more than 20°, a child is considered to have an out-toeing gait. And FPA can influence the distribution of the plantar pressure. Rosenbaum [26] found that in-toeing increasingly loads the lateral aspects of the midfoot and forefoot by as much as 61% and 49%, respectively, whereas out-toeing intensifies the load on the medial aspect, i.e., predominantly the medial midfoot and medial forefoot by as much as 72% and 52%. Traditionally, a flat foot can also be diagnosed by measuring the area of contact between the foot and the ground [22, 27]. However, as far as we are concerned, there is no related low-cost system for pathological gait recognition of children automatically for ordinary parents with only pressure information. The contributions of this study are the following:

1. A low-cost pathological gait-recognition system (PGRS) with an 8 × 8 pressure sensor array is built. With this system, parents can watch children’s gait state daily without the need to consult a podiatrist frequently in hospital. This not only reduces the worry and anxiety of parents, but also improves the efficiency of podiatrists, reducing the pointless counseling for childhood gait.
2. A highly accurate and fast intelligent gait-recognition method (IGRM) is realized in static and dynamic situations utilizing only plantar-pressure data.
3. The effects of the algorithm with plantar pressure data collected in both static and dynamic sections are compared. And the research shows that the IGRMs in the dynamic section have higher average accuracy than those in the static section.

## Methods

**Fig. 1:**
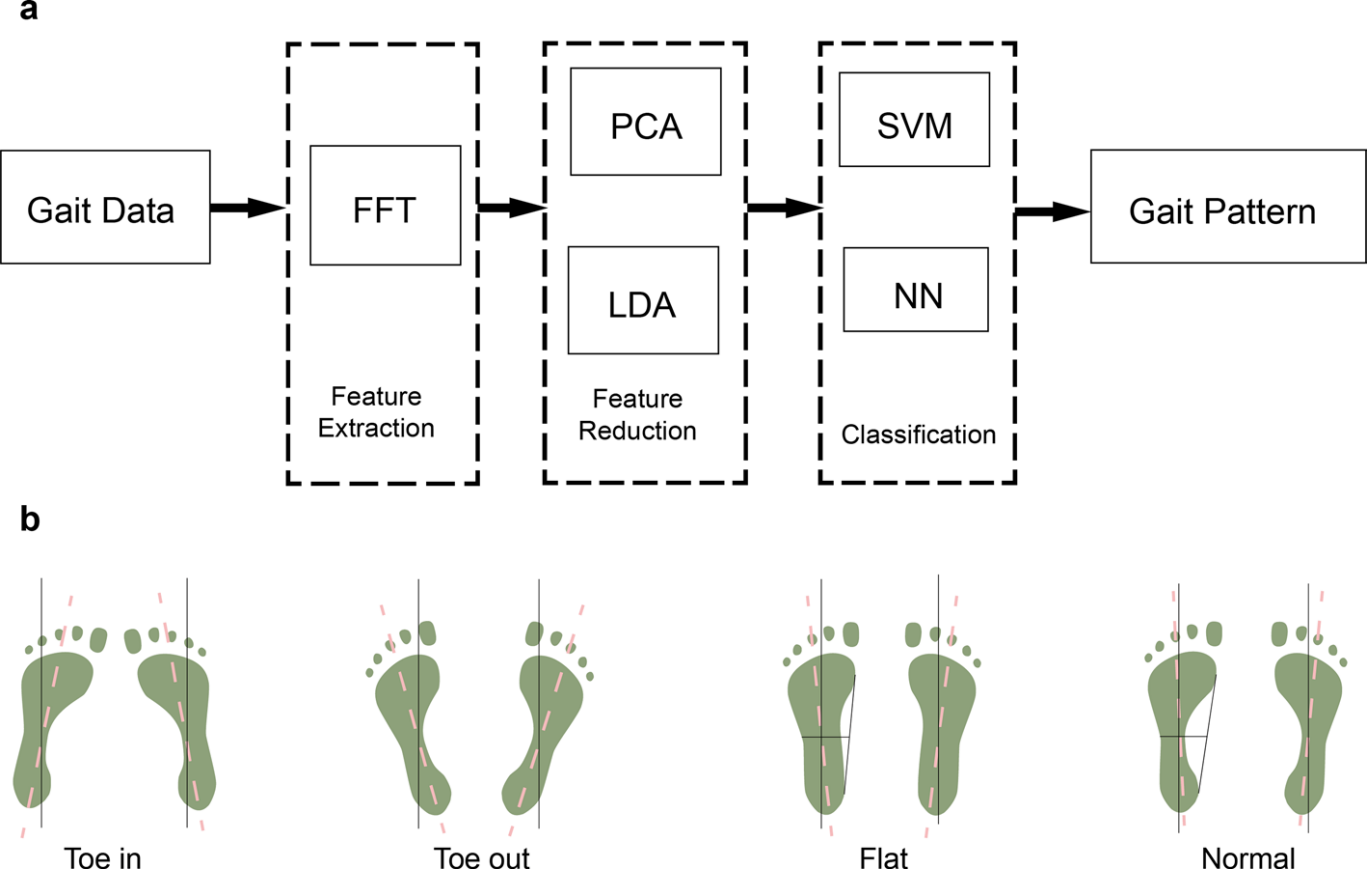
**a** Diagram of the gait-recognition algorithm. **b** Gait pattern for classification—toe-in, toe-out, flat and normal

To deal with the gait data with high dimensionality, temporal dependence, high variability, correlations between curves, and non-linear relationships [4], the proposed gait-recognition algorithm has three steps: feature extraction, feature reduction, and classification. Figure 1 shows an overview of the abnormal gait-recognition algorithm and corresponding gait patterns. Firstly, the data are transformed from time domain to orthogonal domain to handle the gait’s temporal dependence and get data features. Here, several transformations can be used, for example, fast Walsh transform (FWT), discrete cosine transform (DCT), and fast Fourier transform (FFT), among which FFT, used in the proposed IGRM, is the most common and effective one [17, 28–33]. After feature extraction by FFT, feature combination and feature reduction algorithms, PCA and LDA, are done to make gait-data dimensionality lower to apply it in real-time situations. Finally, to handle the high-variability problem, correlations between curves, and non-linear relationships, a robust classification algorithm is used.

### Gait feature extraction from gait cycle

**Fig. 2:**
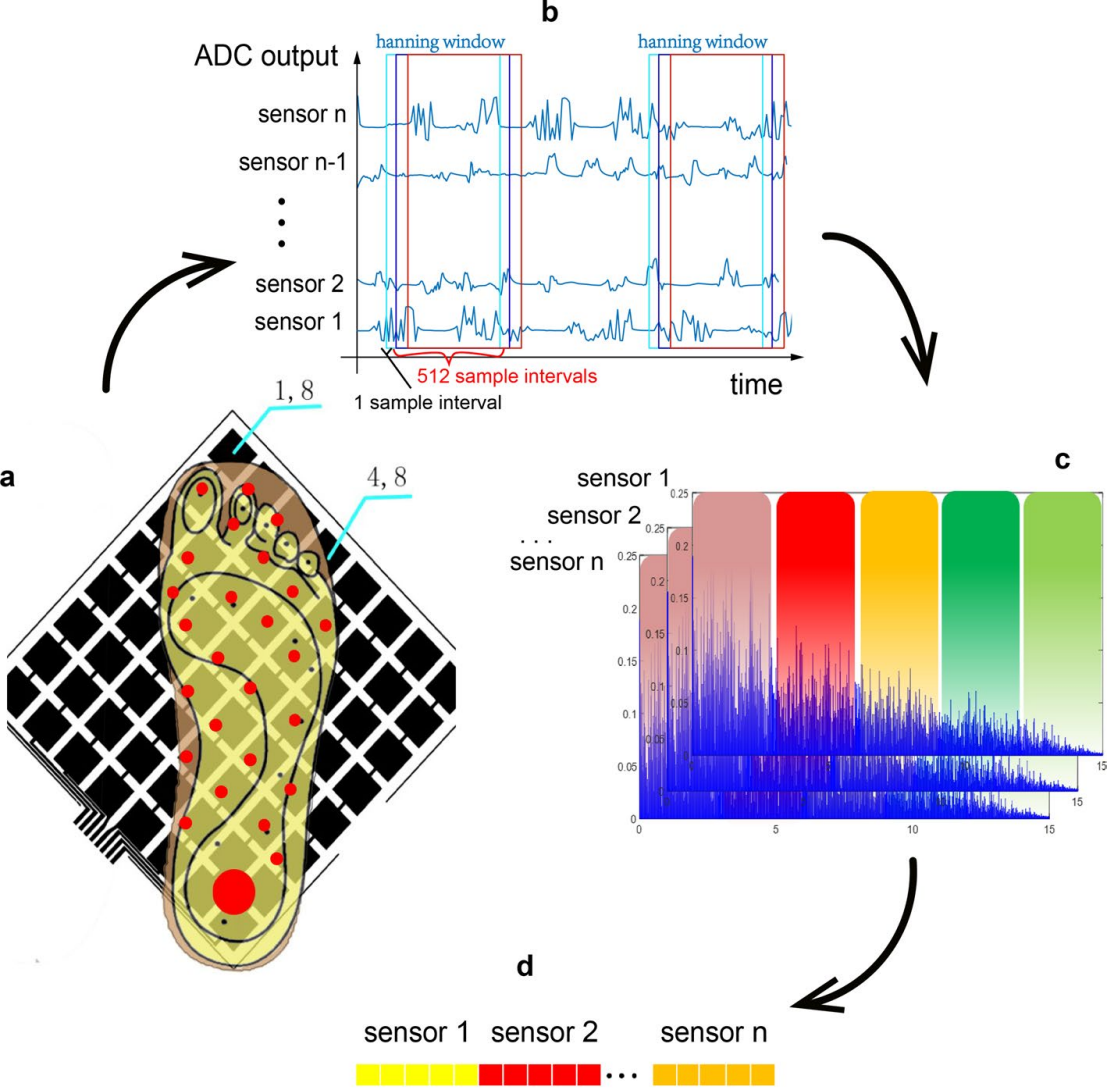
The procedure of gait feature extraction from gait cycle. **a** 30 sensor blocks with the red mark are selected for feature extraction. **b** Sliding window method (Hanning window with 512-sample intervals width) is applied to all n sensor blocks and transformed to frequency domain later. **c** Dividing five frequency bands from FFT frequency spectrum, 0 (exclude)–2 Hz, 2 (exclude)–4 Hz, 4 (exclude)–6 Hz, 6 (exclude)–8 Hz, and 8 (exclude)–10 Hz, and summing all the frequent value in each frequency band to generate a five-elements vector for each sensor. **d** Joining all the five-element vector from each sensor together and normalizing to a 150-element unit vector

In the dynamic situation where subjects walk naturally on the level ground wearing PGRS, **t**he detailed gait feature-extraction process is shown in Fig. 2. For length-*L* data, the *k*th feature of the FFT $S(k)$ can be obtained through

1 $$ S(k) = \sum\limits_{{n = 0}}^{{L - 1}} {t(n)W^{{kn}} } , $$

where k is the integer between 0 (included) and L-1 (included); $W$ is $e^{{ - j2\pi /L}}$; *n* is the time series and $t(n)$ $t(n)$ represents the data value at time *n*.

Simple data sampling strategy with the sliding window method is used to make a proper classification on the assumption that the stochastic process related to the features is stationary over the window interval. We find that if a window interval *L* is chosen wide enough to include multiple gait periods, then the assumption can be acceptable. Consequently, in this step, the temporal dependence is removed through FFT. In this paper, the sliding window is Hanning window with a width of 512 sample intervals (50 Hz sample frequency, corresponding to about 10 gait periods). A 512-length vector $S(k)$ can be got by transforming the plantar-pressure data in the sliding window to frequency domain using FFT. The vector denotes the energy information distribution on the frequency domain of a pressure sensor block during walking. Therefore, vectors $S_{{i,j}} (k)$, where $i$ = 1…8 and $j$ = 1…8, including all 8 × 8 sensor blocks’ frequency information can describe the entire foot energy information distribution. Since the experiments show that the major information of children’s gait data is between 0 and 10 Hz, gait data are divided into five groups which are 0 (exclude)–2 Hz, 2 (exclude)–4 Hz, 4 (exclude)–6 Hz, 6 (exclude)–8 Hz, and 8 (exclude)–10 Hz. This division of groups is optimized by conducting repeatedly the experiments with different number of groups to get the best performance of prediction accuracy, prediction accuracy variation, prediction time cost on the overall classification task. Summing all of the amplitude of frequency components in each group, the 512-length vector $S(k)$ is transformed to a five-element feature vector. To denote the information of the entire foot, 30 five-element feature vectors from 30 sensors are joined together, obtaining a 150-element vector as a training sample. Note that bodyweight is different for different children, the 0 Hz pressure information is excluded and the 150-element training sample is normalized to a unit vector to eliminate the weight information influence when classifying the foot types.

In the static situation where subjects stand naturally on the level ground wearing PGRS, the 0 Hz pressure information is kept only and the feature number of a plantar pressure sensor is no longer five but one. The final training sample for gait recognition becomes a 30-element vector. Normalization is also used to vanish the influence of the bodyweight of the subjects.

### Feature reduction

**Fig. 3:**
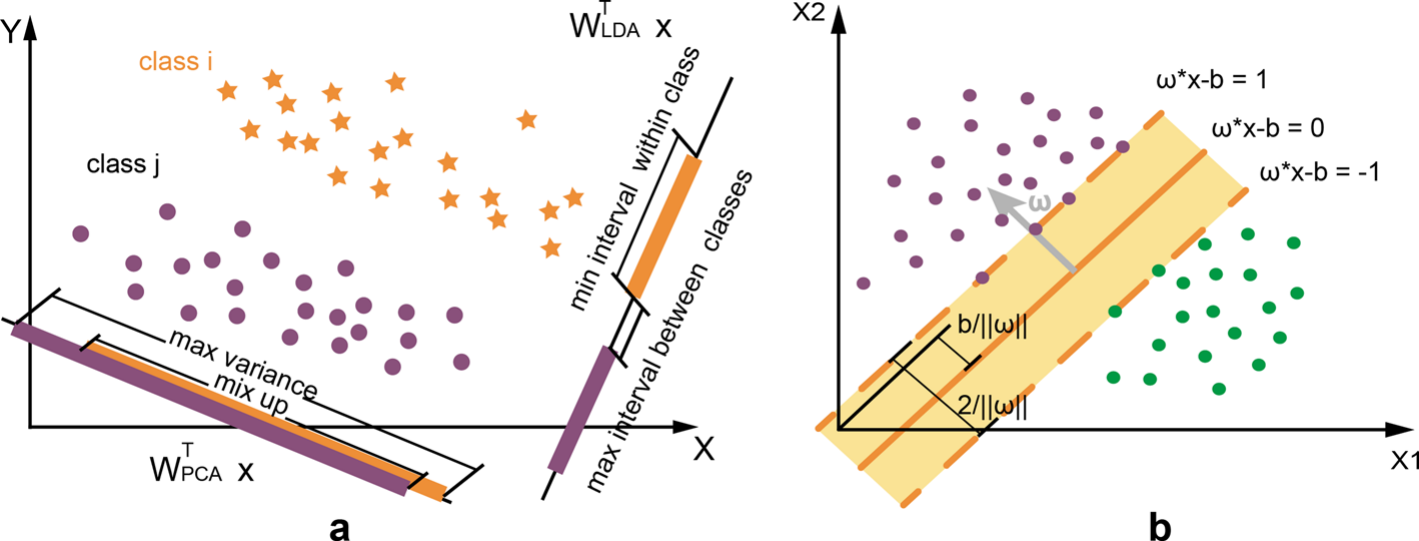
**a** LDA and PCA algorithm performance in classification in the specific situation. In this situation, LDA’s performance is better than PCA’s. **b** SVM classification. The main idea of the SVM is projecting data points into a higher dimensional space, specified by a kernel function, and computing a maximum-margin hyperplane decision surface that separates the two classes

In this step, plantar-pressure information is further compressed and keeps effectiveness at the same time. As a quite effective and common unsupervised method in signal processing, PCA keeps the variance of the original data during the dimension reduction. However, PCA may cause a mix-up of different-label data in some situations, as Fig. 3a shows. In these situations, another supervised method, LDA, is more efficient. LDA maximizes the average differences among class projections while minimizing average projections of each class (intraclass) after feature reduction. In mathematics, the main idea of LDA can be described as maximizing $J\left( W \right)$:

2 $$ \max J\left( W \right) = \frac{{\sum\limits_{i} {\tilde{S}_{{B\left( i \right)}} } }}{{\sum\limits_{j} {\tilde{S}_{{W(j)}} } }} = \frac{{\sum\limits_{i} {{\mathbf{W}}^{T} S_{{B\left( i \right)}} {\mathbf{W}}} }}{{\sum\limits_{j} {{\mathbf{W}}^{T} S_{{W(j)}} {\mathbf{W}}} }}, $$

where $S_{B}$ and $S_{W}$ are the dispersion between two different classes and within a class, respectively; i and j represent the class number; symbol $\sim$ represents the variable after dimensional reduction and ${\mathbf{W}}^{T}$ is defined as the corresponding transformation matrix.

**Fig. 4:**
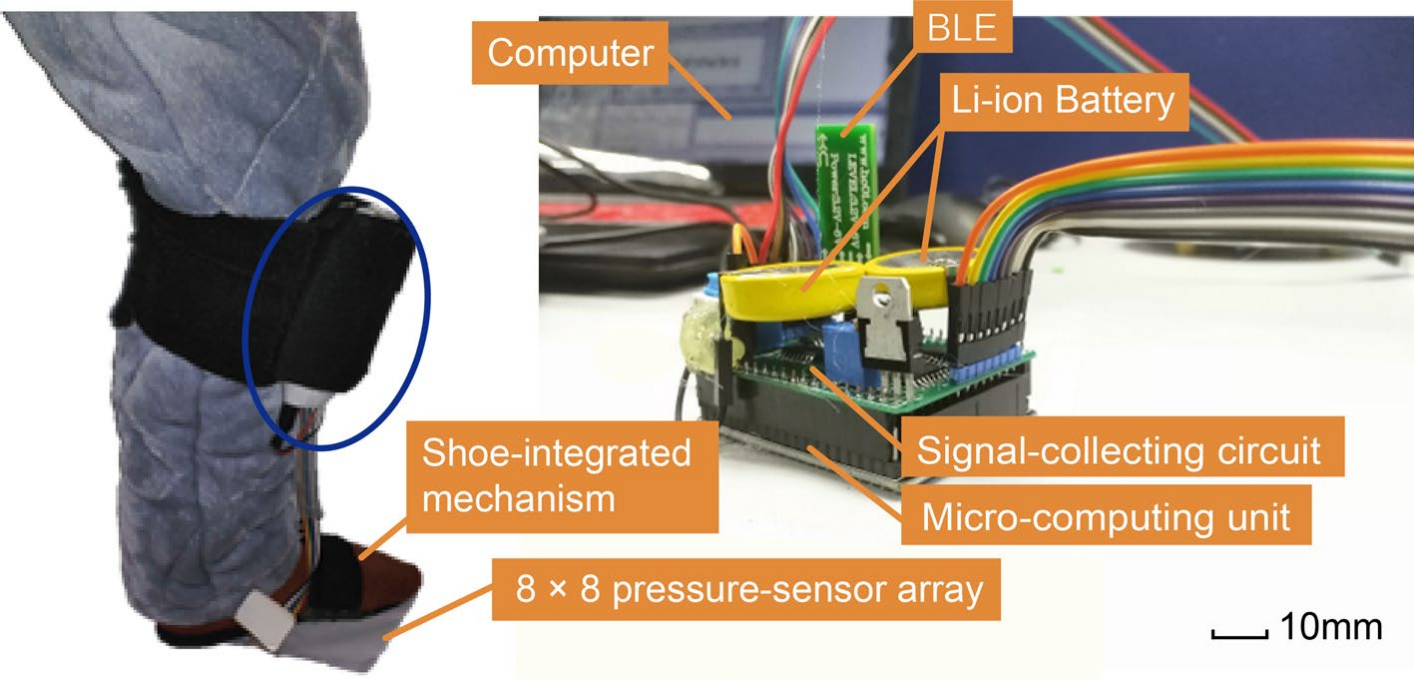
Pathological gait-recognition system. 8 × 8 sensor array is under the slipper and controller attached to the child’s leg with hook-and-loop fasteners. The control circuit board contains a signal-collecting circuit, a low-energy Bluetooth device (HC-42 with Bluetooth 5.0, HuiCheng Information Technology Co., Ltd., China), STM32F103 controller, and two 4.2-V Li-ion batteries. The signal-collecting circuit operates with 5 V of power generated by an LM7805 unit (KIA7805AP, three-terminal positive voltage regulator of 5 V, KEC, China) and the STM32F103 circuitry operates with 3.3 V generated by an AMS117 unit (low-dropout-voltage regulator with fixed 3.3 V, Advanced Monolithic Systems, Inc.)

### Gait-classification model

Our goal in this step is to classify the gaits into different classes, i.e., toe-in, toe-out, flat, and normal according to the feature vectors. Many algorithms in the machine learning field can be used for binary classification problems. In this paper, NN and SVM are used to perform the gait-classification function.

As many researchers have proved, SVM can work very well for multi-dimensional data[34, 35]. More importantly, sma3ll computation is needed for the final decision function of SVM which comprised only a few support vectors. As shown in Fig. b, it obtains a hyperplane, ${\mathbf{\omega }}^{{\mathbf{T}}} {\mathbf{x}} + b{\text{ = }}0$, to classify two classes by maximizing the margin to the closest data from two classes separately. The decision function can be described as

3 $$ \left\{ {\begin{array}{*{20}c}    {f({\mathbf{x}}) = {\mathbf{\omega }}^{{\mathbf{T}}} {\mathbf{x}} + b}  \\    {y = {\text{sign}}(f({\mathbf{x}}))}  \\   \end{array} } \right., $$

where ${\mathbf{x}}$ is the feature vector, ${\mathbf{\omega }}$ is the normal vector to the hyperplane, $\frac{b}{{\left\| {\mathbf{\omega }} \right\|}}$ determines the offset of the hyperplane, and $y$ are either 1 or −1, each indicating the class to which the feature vector belongs. A linear SVM’s goal is to minimize the risk function:

4 $$ \begin{gathered}   \begin{array}{*{20}c}    {\mathop {{\text{Minimize}}}\limits_{{\omega ,b}} } & {\frac{1}{2}}  \\   \end{array} {\mathbf{\omega }}^{{\mathbf{T}}} {\mathbf{\omega }} + C\sum\limits_{{i = 1}}^{N} {\xi _{i} }  \hfill \\   {\text{Subject to }}\left\{ \begin{gathered}   \xi _{i}  \ge 0 \hfill \\   y_{i} \left( {{\mathbf{\omega }}^{{\mathbf{T}}} {\mathbf{x}} + b} \right) \ge 1 - \xi _{i}  \hfill \\  \end{gathered}  \right., \hfill \\  \end{gathered} $$

where the term ${\mathbf{\omega }}^{{\mathbf{T}}} {\mathbf{\omega }}$ ${\omega }^{T}\omega$ is called the regularization term and $C\sum\limits_{{i = 1}}^{N} {\xi _{i} }$ is empirical tolerance [36]. Empirical tolerance is not only used to remove data noise, but also to deal with data non-linearly separable. To obtain the linear inseparable classification model, the penalty parameter $C$ calculates the penalties for errors by determining the trade-off between the empirical tolerance and regularized term. The larger $C$ is, the stronger penalties are assigned to errors.

To solve the non-linear classification problem effectively, a kernel function is introduced to the decision function:

5 $$ f({\mathbf{x}}^{{\mathbf{T}}} {\mathbf{x}})\xrightarrow{{{\text{kernel}}}}f(\phi \left( {{\mathbf{x}}_{{\mathbf{i}}} {\mathbf{,x}}} \right)), $$

where $\phi \left( {{\mathbf{x}}_{{\mathbf{i}}} ,{\mathbf{x}}} \right)$ is a kernel function that maps ${\mathbf{x}}$ $\boldsymbol{x}$ space to a higher-dimensional space so that a hyperplane can be found to classify samples. In this paper, linear or RBF kernel is used in our classification model. To solve this formulation, SVM can be trained by Platt's sequential minimal optimization (SMO) algorithm [37].

NN is a network of neurons and the connections of biological neurons are modeled as weights. Training the weight using the backpropagation algorithm, NN can learn the mapping relationship between input and output. Regardless of its powerful ability for solving non-linear problems, NN is a black box comparing with explainable and intuitive SVM.

## Results

In this part, a low-cost PGRS is built to measure children’s plantar-pressure data during walking, and children's experiments in dynamic and static sections are designed to verify the performance of recall, precision, and time cost of the IGRM. Several typical gaits, namely, toe-in, toe-out, flat, and normal, are involved. All of the subjects signed informed consent forms before experiments.

### Pathological gait-recognition system

As shown in Fig. 4, a PGRS consists of an 8 × 8 pressure-sensor array, a signal-collecting circuit, a micro-computing unit, and a wearable shoe-integrated mechanism is designed.

**Fig. 5:**
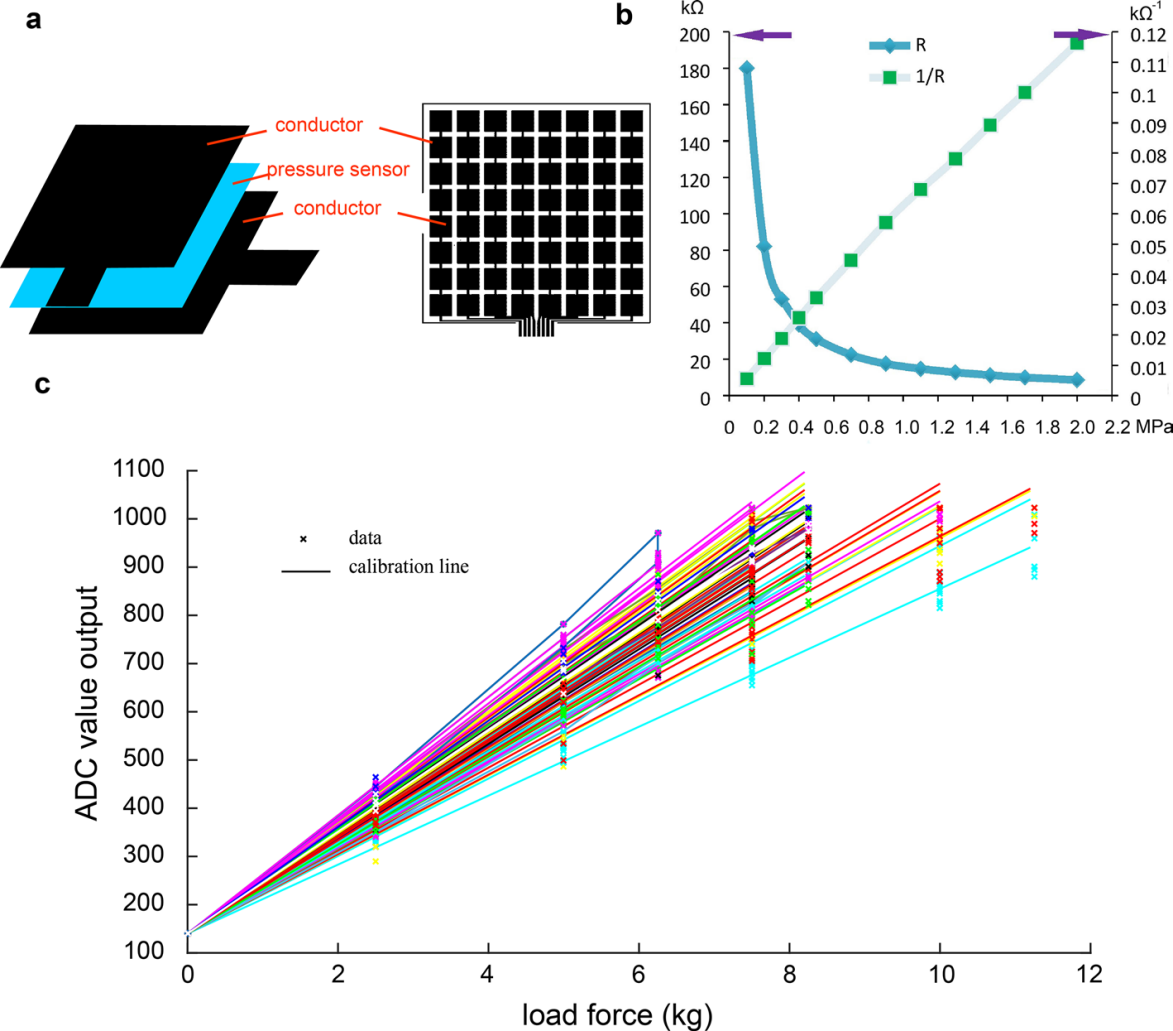
**a** The three-layer structure of the piezoresistive sensor. **b** Piezoresistive sensor pressure–resistor characteristic curve. Horizontal axis represents the pressure loaded on the sensor and vertical axis is the resistor of sensor. **c** Sensor calibration. Same color points represent a certain sensor block output during calibration and regression straight line with the same color is its result. A total of 64 sensor block calibration results are shown

The plantar pressure of adults is in the range of 0–1000 kPa during walking [38], and the maximal pressure can up to approximately 1400 kPa when doing sports. Based on weight conversion, children's plantar pressure is estimated to be in the range of 0 to 700 kPa. Therefore, the pressure-detection range of the sensor array is chosen as 0–1000 kPa. The 8 × 8 piezoresistive sensor array structure and parameter characteristics of this series (Changzhou Roxi Electronic Technology Co. LTD, China) are shown in Fig. 5a and b. The relationship of load pressure $P$ $P$ onto the sensor and its resistance $R_{x}$ ${R}_{x}$ can be described as:

6 $$ R_{x}  = \frac{1}{P}K_{{p - r}} , $$

where $K_{{p - r}}$ is the sensor characteristic constant.

In sensor calibration process, a set of standard weights are used to load on the sensor block. The load is added from 0 to 11 kg and then reduced from 11 to 0 kg for each sensor block twice, and linear regression was applied to the data by least square method to get the calibration line. All the test results of the 8 × 8 sensors’ performance using the control circuit mentioned below are shown in Fig. 5c, indicating its good repeatability and linearity.

**Fig. 6:**
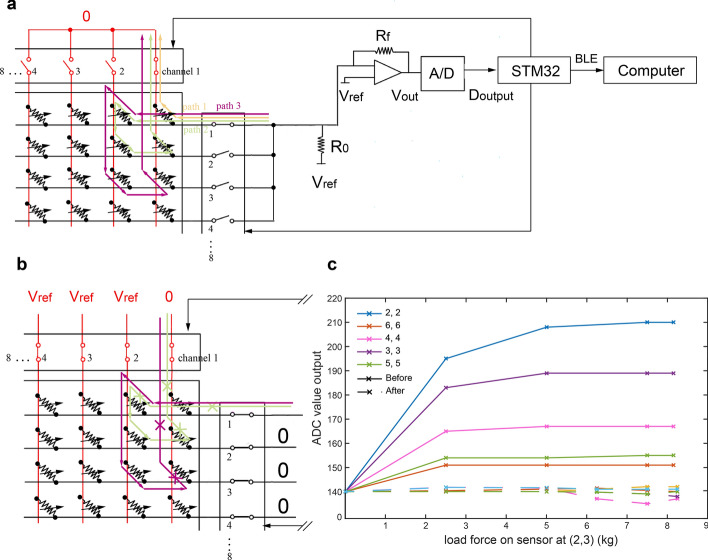
Piezoresistive sensor array scanner electronic schematic. **a** Schematic before applying elimination crosstalk method. A microcontroller is used to select the sampling row and column channel by controlling quad bilateral switch CD4066 chip, while other unselected channels are remaining high resistance. Crosstalk output is found between sensor blocks as path 1 and path 2 show. **b** Schematic after applying elimination crosstalk method. When a certain row and column channels are selected (row channel 1 and column channel 1 is selected here), we pull down other row channels to ground and pull up other column channels to Vref, such that path 2 and path 3 will be cut off and path 1 will remain. Crosstalk output between sensor blocks can be eliminated. **c** The load force on the sensor block at (2,3) has less influence on other sensors' value output after sensor array applying elimination crosstalk. The dotted line represents data after sensor array applying elimination crosstalk and the solid line represents data before sensor array applying elimination crosstalk

For the signal-collecting circuit and the micro-computing unit, as the schematic is shown in Fig. 6a, a microcontroller (STM32F103C8T6 with Cortex-M3 core and a maximum CPU speed of 72 MHz; STMicroelectronics Corp., USA) was chosen as its control core. Noninverting amplifier converts resistor of the sensor $R_{x}$ to voltage $V_{{out}}$. The charging resistor $R_{0}$ used to stable the ADC output is 1 KΩ [39]. We use two pieces of quad bilateral switch HCF4066B chip (STMicroelectronics Corp., USA) to compose one 8:1 analog switch. The state of the switch is controlled by the logic level of I/O from the microcontroller. The microcontroller is programmed to select the sensor block one by one using two 8:1 analog switches and to read the sensor block’s output. Two 4.2-V Li-ion batteries provide power, generating 5 V with LM7805 (KIA7805AP, three-terminal positive voltage regulator of 5 V, KEC, China) for the signal-collecting circuit and 3.3 V with AMS117 (low-dropout-voltage regulator with fixed 3.3 V, Advanced Monolithic Systems, Inc.) for the microcontroller.

**Fig. 7:**
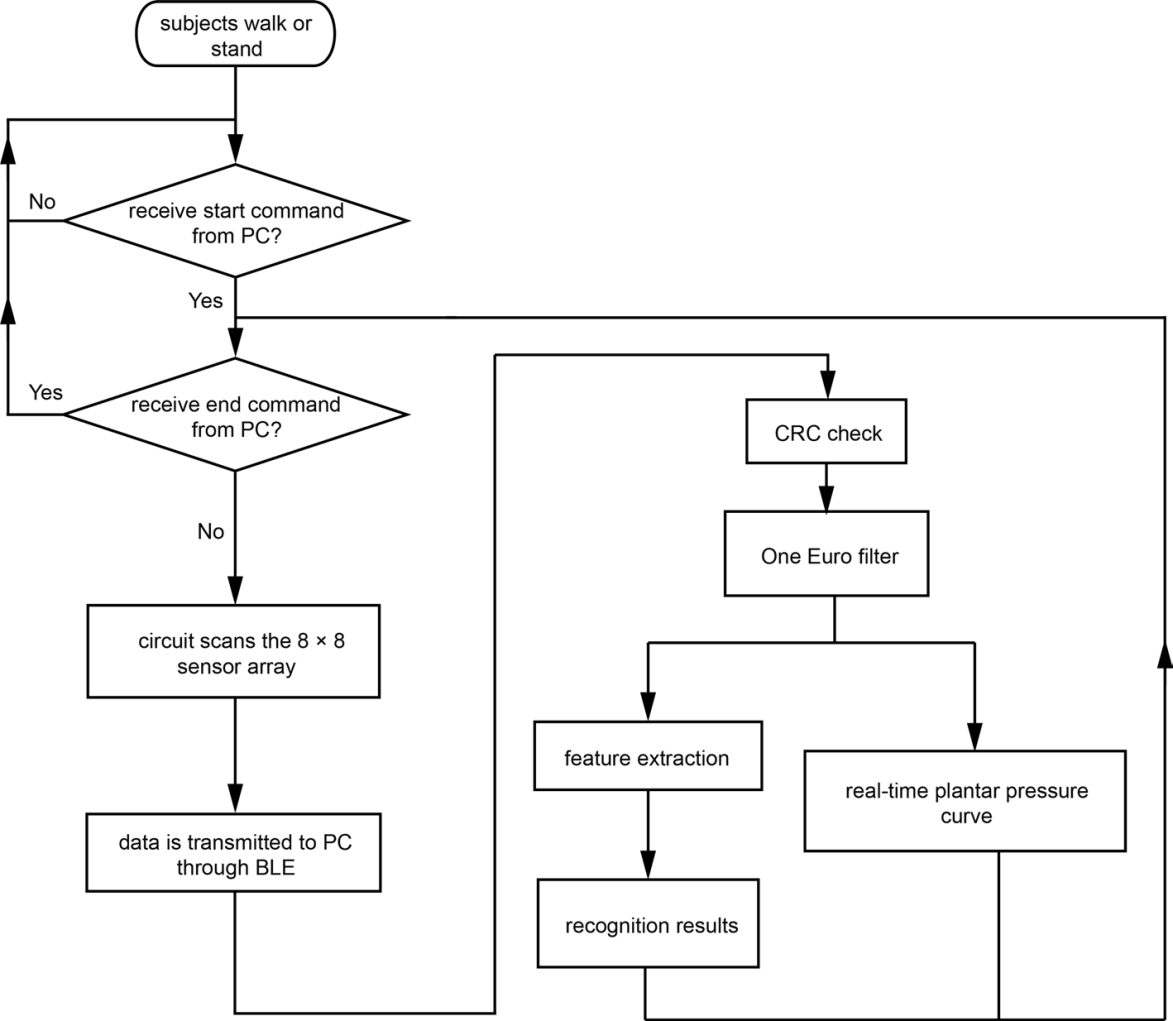
GUI background flowchart. GUI/PC can receive commands from people in the GUI and use BLE to communicate with the controller system

**Fig. 8:**
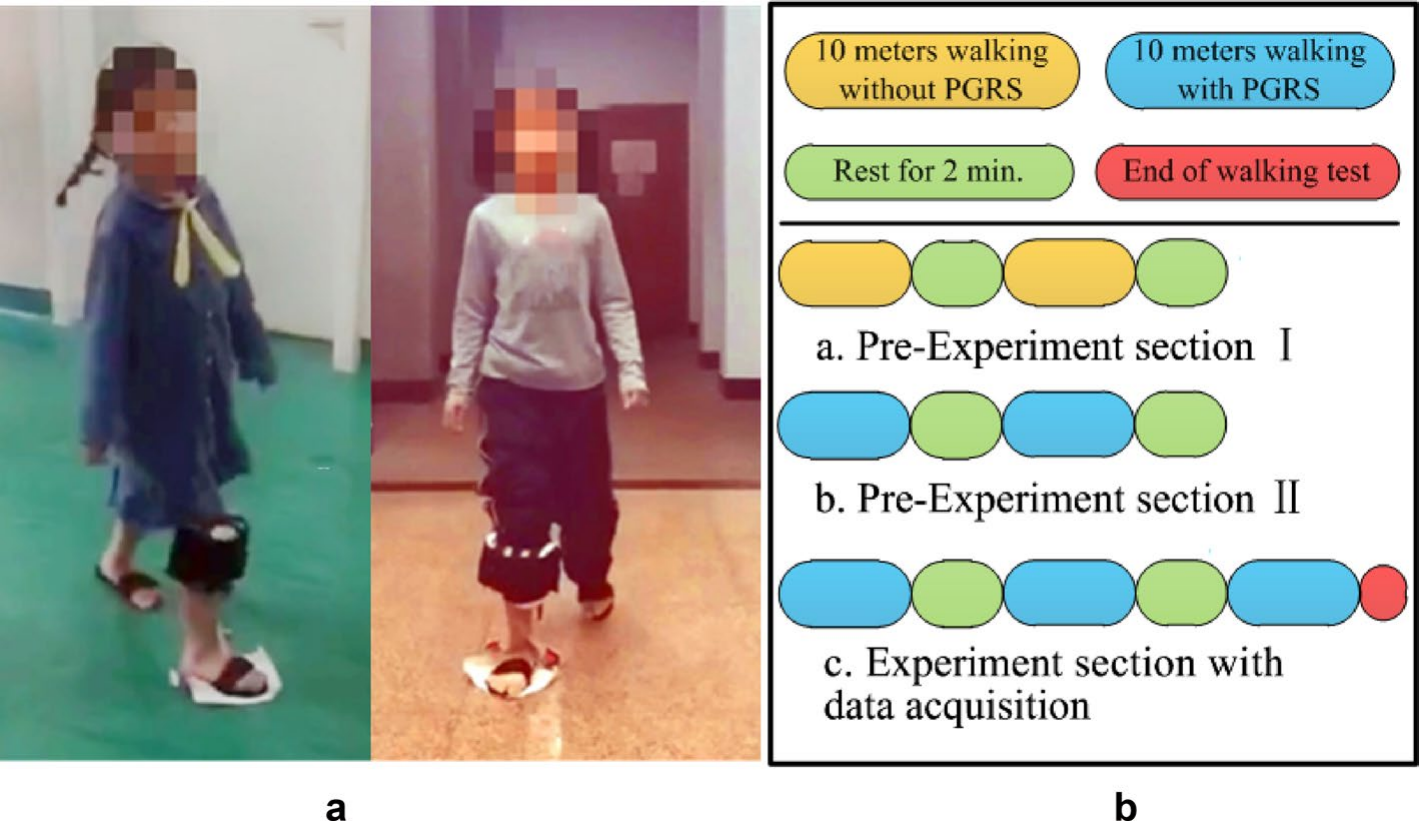
Experiment in the dynamic section: **a** snapshots of subjects; **b** experimental procedure of the dynamic section

**Fig. 9:**
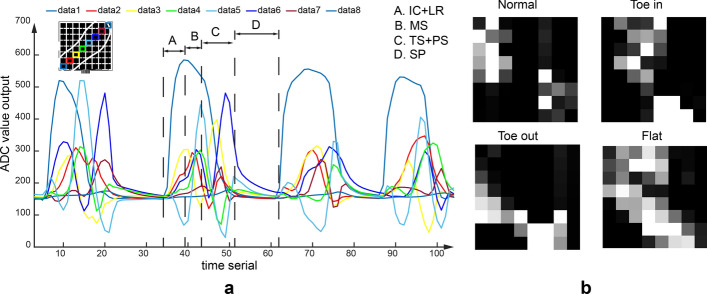
Plantar-pressure data acquisition results. **a** Curve of plantar pressure with time during level-ground walking in the dynamic-section experiment. Subfigure on the left top shows the placement of the sensor corresponding to data with the same color. **b** Typical toe-in, toe-out, and normal foot-pressure distribution in the static section experiment. The plantar pressure value is expressed in different grey scales. Pure black means zero pressure, pure white means the largest pressure

**Fig. 10:**
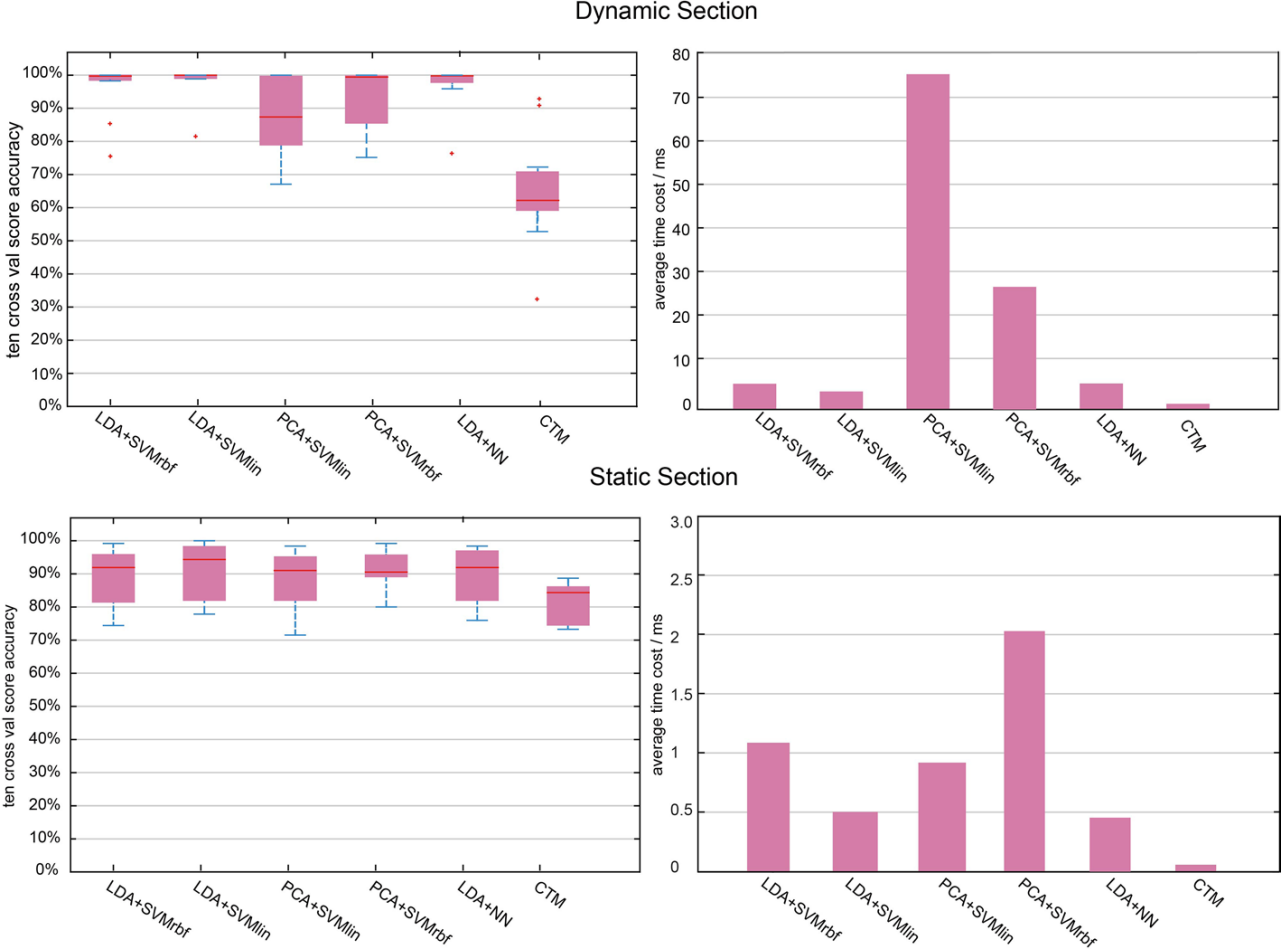
Accuracy and time cost results of IGRM in the dynamic and static experiments. The x-axis label means reduction algorithm + classification algorithm. For example, LDA + SVMlin means that IGRM’s feature reduction algorithm is LDA, and SVM with linear kernel is its classification algorithm. Here, PCA components are 7 (capture 90% of the variance) in the dynamic section and 12 (capture 90% of the variance) in the static section. And the dimension of LDA is 3 in both dynamic and static section

**Fig. 11:**
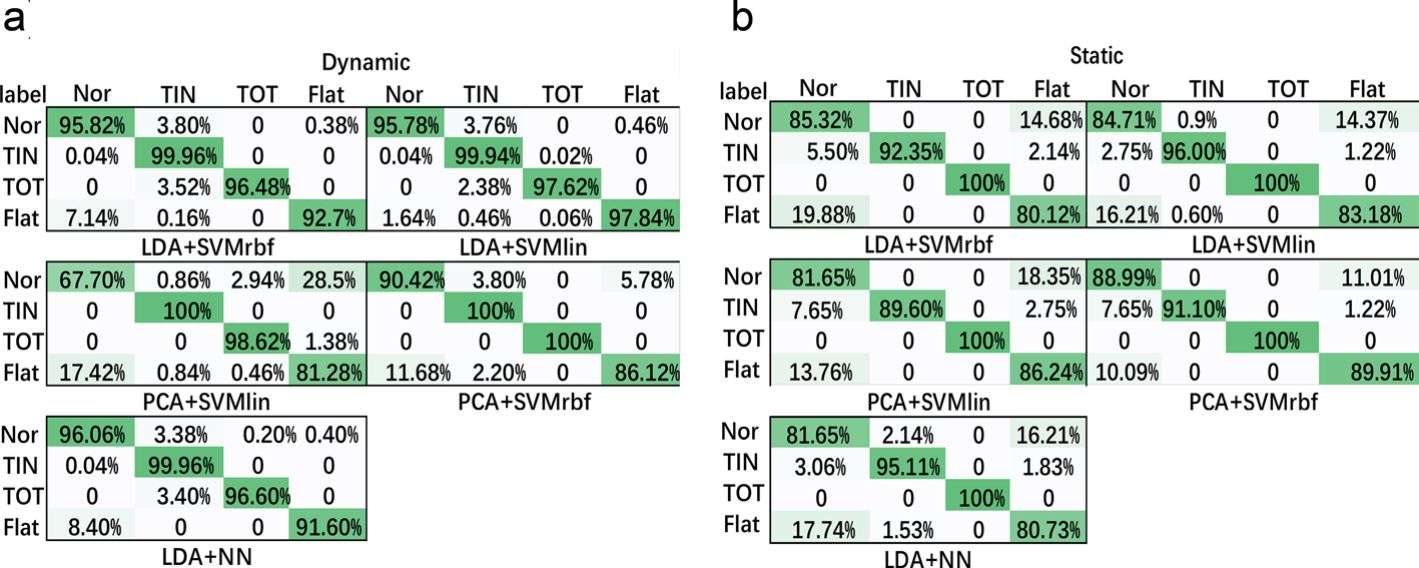
Average confusion matrix of algorithm results in tenfold cross-validation of **a** dynamic and **b** static sections. The labels on the column are predicted labels and those on the row are actual labels; the samples number at the row label i and the column label j means the average number of times instances of class i are classified as class j in the tenfold procedure

Through an amplifier and feedback resistance $R_{f}$ ${R}_{f}$, the output voltage $V_{{out}}$ of the amplifier is linear to the pressure sensor received. Defining $D_{{{\text{output}}}}$,$V_{{{\text{adc\_ref}}}}$ and $m$ as the digital value received by the analog-to-digital converter (ADC), the reference voltage and resolution of the ADC, respectively, $V_{{out}}$ ${V}_{out}$ is related to the resistance of a specific sensor. We have

7 $$ V_{{out}}  = \frac{{R_{x}  + R_{f} }}{{R_{x} }}V_{{ref}} , $$

8 $$ D_{{{\text{output}}}}  = 2^{m} \frac{{V_{{{\text{out}}}} }}{{V_{{{\text{adc\_ref}}}} }} = 2^{m} \left( {\frac{{k_{{P - R}}  + P \times R_{f} }}{{k_{{P - R}} }}} \right)\frac{{V_{{{\text{ref}}}} }}{{V_{{{\text{adc\_ref}}}} }}, $$

where $V_{{{\text{adc\_ref}}}}$ is 5 V, $V_{{ref}}$ is 0.5 V and *m* is 10 in the experiments, so that the load pressure $P$ can be reflected by $D_{{{\text{output}}}}$.

During the test, it was found that the pressure on one sensor would lead to an unexpected output of the rest of the sensors. Crosstalk is found between sensor blocks caused by current path 2 and path 3 when measuring the sensor value at (1,1), where (No. 1, No. 2) means the sensor block at row channel No. 1 and column channel No. 2, as shown in Fig. 6a. The closer the distance between two sensors is, the stronger their interactions are. To extract individual sensor resistor value one after another from the 8 × 8 sensor array and eliminate the crosstalk between each sensor, as shown in Fig. 6b [40, 41], the active sensor during readout was selected by grounding one of the 8:1 analog switch channels on the column, while other channels are pulling up to $V_{{{\text{ref}}}}$ ${V}_{ref}$. The same control operation is applied to row pins of the 8 × 8 sensor array at the same time, so that the current path except path 1 such as path 2 will be cut off. The sampling result shown in Fig. 6c indicates that the ADC output value (maximum is 1024) of a certain sensor influenced by other sensors is within 10 (32.2 mV output, corresponding to 0.282 kg loaded), which is much better than the previous maximum, almost 70 (225 mV output, corresponding to 1.977 kg loaded).

### Gait-pressure data collection

A GUI-based program using MatLab R2016a (MathWorks, USA) was designed to make the pressure information more available and more visual. Its flowchart is shown in Fig. 7 , which has functions of filtering the pressure data, showing the real-time pressure data curve of a specific plantar-pressure sensor, saving pressure data in the computer, and obtaining the gait-pattern results.

To meet the expected situation where people normally want to have low disturbance in slow signals and low lag in fast signals, the filter used in the aforementioned program is One Euro filter [42]. And [41] has found that a straightforward linear relationship between cutoff frequency and the absolute speed of the input signal works well. To construct One Euro filter, the frequency spectrum information of input is studied. Based on the experiment data collected at the Children's Hospital, Zhejiang University School of Medicine, the frequency spectrum of plantar-pressure data of the children shows almost all information is located in 0–10 Hz, which is similar to the features found by Hangqi Wei [43] about adult plantar pressure.

As shown in Table 1, 17 subjects who had undergone gait diagnosis by a specialist were recruited to participate in the experiment. The experiments are divided into two sections: dynamic and static section. The experimental procedures of the dynamic section are shown in Fig. 8. Subjects are asked to walk naturally for 10 m two times with a 2-min gap on level ground. In the static section, children are expected to stand naturally and comfortably for about 10 s five times.

One example of the collected plantar-pressure data in the dynamic section is shown in Fig. 9a. The plantar-pressure curve can be divided into several gait phases: initial contact (IC), loading response (LR), mid-stance (MS), terminal stance (TS), pre-swing (PS), and swing phase (SP). And typical toe-in, toe-out, and normal foot-pressure distribution in static section is shown in Fig. 9b.

### Gait-recognition results

The IGRM is coded in Python with scikit-learn library. PCA or linear discriminant analysis (LDA) is used to reduce plantar-pressure data dimension in both dynamic and static sections. Coefficient gamma of RBF kernel in SVM is chosen as 1/(features × variance of data) and penalty parameter C is 1. The layer of the NN is 3 and the neuron number of the hidden layer is 10. The total experiment samples are 20,000 in which normal, toe-in, toe-out, and flat have 5000 samples, respectively, in the dynamic section and 1308 in which normal, toe-in, toe-out, and flat have 327 samples, respectively, in the static section. The scheme of evaluation included stratified tenfold cross-validation with recall, precision, and a time cost as metrics. All statistical analysis was performed using SPSS version 22 (SPSS Inc., Chicago, IL, USA). the time cost is calculated by predicting 2000 samples in the dynamic section or 130 samples in the static section using the same computer.

Experimental performances of dynamic and static sections, respectively, are shown in Tables 2 and 3. Figure 10 shows the distribution of the accuracy of different types of algorithms which are evaluated by tenfold cross-validation and the average time cost per fold. The classification result of classification models to all the samples is shown in the average confusion matrix in Fig. 11.

**Table 1:** Experimental subjects’ basic information

Index	Age (years)	Diagnosis	Male/female
1	6	Flat	Female
2	10	Flat	Female
3	8	Flat	Male
4	9	Flat	Male
5	10	Flat	Female
6	7	Flat	Female
7	9	Flat	Male
8	9	Flat	Male
9	10	Flat	Male
10	8	Normal	Female
11	5	Normal	Female
12	6	Normal	Male
13	6	Normal	Female
14	8	Toe-in	Female
15	9	Toe-in	Male
16	8	Toe-out	Female
17	9	Toe-out	Male

**Table 2:** Experimental results data of dynamic section

Algorithm	Recall	Average precision (%)	Time cost (ms)	Accuracy/time cost *P*-value
LDA + SVMrbf	0.96/1.00/0.96/0.93	96.24	6.10 4.40 75.20 27.40 5.10	0.991 / < < 0.01
LDA + SVMlin	1.00/1.00/1.00/1.00	97.79		–
PCA + SVMlin	0.68/1.00/0.99/0.81	87.24		0.007 / < < 0.01
PCA + SVMrbf	0.90/1.00/1.00/0.86	94.13		0.107 / < < 0.01
LDA + NN	0.96/1.00/0.97/0.92	97.38		0.702 / 0.064

“–” means the *P*-value of this column is computed based on this algorithm. In the ***Recall*** column, the value separated by “/” means recall value of normal, toe-in, toe-out, and flat, respectively, from left to right. In the ***Accuracy/time cost P-value*** column, the value separated by “/” means *P*-value of prediction accuracy and *P*--value of time cost *P*--value between algorithm on this row and PCA + SVMlin, respectively, from left to right

**Table 3:** Experimental results data of static section

Algorithm	Recall	Average precision (%)	Time cost (ms)	Accuracy/time cost *P*-value
LDA + SVMrbf	0.85/0.92/1.00/0.80	89.39	1.10	0.698/ < < 0.01
LDA + SVMlin	0.85/0.96/1.00/0.83	90.90	0.50	–
PCA + SVMlin	0.82/0.90/1.00/0.86	89.49	0.89	0.690/ < < 0.01
PCA + SVMrbf	0.89/0.91/1.00/0.90	92.41	2.07	0.623/ < 0.01
LDA + NN	0.82/0.95/1.00/0.81	89.75	0.46	0.765/0.388

“–” means the *P*--value of this column is computed based on this algorithm. In the ***Recall*** column, the value separated by “/” means recall value of normal, toe-in, toe-out, and flat, respectively

According to Table 2, in the dynamic section, LDA + SVMlin reaches the highest average accuracy, 97.79%. In the term of accuracy, independent t-test shows a significant difference between LDA + SVMlin and PCA + SVMlin (*P*-value = 0.007). There is no significant accuracy difference among LDA + SVMrbf, PCA + SVMrbf, and LDA + NN. However, LDA + SVMlin, 4.40 ms per 2000 samples, has a significant smaller time cost than LDA + SVMrbf (*P*-value <  < 0.01), PCA + SVMlin (*P*-value <  < 0.01), and PCA + SVMrbf (*P*-value <  < 0.01). PCA + SVMlin has the largest time cost (*P*-value <  < 0.01), 75.2 ms per 2000 samples. In the static section, PCA + SVMrbf reaches the highest average accuracy, 92.41%. However, PCA + SVMrbf has a significantly larger time cost than others (*P*-value <  < 0.01), 2.07 ms per 130 samples. Compared with other algorithms, LDA + SVMlin has no significant difference with other algorithms in terms of accuracy, but it has a significantly smaller time cost (*P*--value <  < 0.01) than LDA + SVMrbf, PCA + SVMlin, and PCA + SVMrbf.

Taking precision, time cost, and statistical analysis into consideration, LDA + SVMlin and LDA + NN are excellent classifiers in both dynamic and static sections.

## Discussion

The results in Fig. 10 prove the feasibility, robustness, and high average accuracy of the proposed approach. All of the IGRMs have been identified with a practically applicable degree of average accuracy either in the dynamic or static section.

From the experimental results above, LDA + SVMlin (97.79% in average precision), LDA + NN (97.38%) are excellent classifiers in both dynamic and static sections. An independent *t*-test shows no significant difference between LDA + NN and LDA + SVMlin in terms of accuracy (*P*-value = 0.702 in the dynamic section, *P*-value = 0.765 in the static section) and time cost (*P*-value = 0.064 in the dynamic section, *P* = 0.388 in the static section). However, based on the good interpretability of SVM, a combination of LDA and SVM with linear kernel is our first choice.

The accuracy distribution of the IGRMs shows that LDA is much better than PCA in pathological gait recognition. As shown in dynamic section of Fig. 10 , LDA + SVMrbf and LDA + SVMlin have a significantly smaller variance than PCA + SVMrbf and PCA + SVMlin in the dynamic section, respectively. However, in the static section, the accuracy of LDA + SVMrbf and PCA + SVMrbf, LDA + SVMlin, and PCA + SVMlin has no significant difference. We infer it might be caused by the fact that plantar pressure data in the dynamic section are more complex and efficient than those in the static section. Besides, LDA has higher efficiency in terms of time cost. Results show that PCA + SVM is approximately two to five times the time cost of the LDA + SVM.

According to our prior knowledge, plantar pressure in the static situation have more information about foot types than that in the dynamic situation. More interestingly, we found that the IGRMs in the dynamic section have higher average accuracy than those of the static section. Combined with the corresponding pressure distribution, it can be inferred that children may act more naturally in the dynamic section than in the static section. Regarding pre-experiment sections I and II, the walking gestures of children resemble an actual situation in the dynamic section, while in the static section children are prone to stand unnaturally when asked to stand still, which causes the plantar pressure collected by PGRS unable to reflect the actual foot type. Meanwhile, according to the opinion of an experienced clinician, it is quite difficult to judge toe-in and toe-out in the static section unless it is in a serious stage.

To test the methodology of generalization performance to unseen participants, a new simulation under static and dynamic conditions, respectively, is conducted. During the simulation, 17 subjects are divided into two sets, training set containing 16 subjects and testing set containing one subject, taking turns to choose a different subject as testing set. As a result, the accuracy of some subjects can reach up above 95% and the variance of prediction accuracy is quite large. The performance of toe-in or toe-out are quite low, less than 50%. Comparatively speaking, the generalization performance of normal and flat are much better. The average accuracy precision of flat and normal is 85.78% and 84.71%, respectively, and the maximum can both reach up above 95% in dynamic section. Corresponding accuracy precision in static section is, 73.48% and 78.10%, respectively, and the maximum can both reach up above 95%. We think it is caused by the small number of subjects and the dataset is not large enough. Due to the lack of patients with toe-in or toe-out, this dataset lets the model falls into subjects’ specific walking patterns, causing the accuracy precision below 50%. With more subjects involved in our dataset, we think the generalization performance could converge to much better results, despite the difficulty to collect enough subjects. However, the experiment also shows the efficiency of our method in children gait monitoring scenarios.

## Conclusions

In this paper, an effective IGRM for pathological gait recognition with dimensional reduction and a classification algorithm is put forward. Also, a low-cost and wearable PGRS with an 8 × 8 pressure sensor array was built. The experimental results show that the proposed IGRM (LDA + SVMlin) has both high accuracy and low prediction time cost in the dynamic section, that is, 97.79% average accuracy and a 4.4-ms prediction time per 2000 samples, while in the static section 90.90% average accuracy and a 0.5-ms prediction time per 130 samples were realized. Additional generalization performance experiment shows IGRM has 85.78% and 78.81% inter-subject recognition accuracy, respectively, in the static and dynamic sections. Another phenomenon found in the experiments is that pathological gait is detected more effectively in the dynamic section since children act more naturally in walking than just standing.

In conclusion, a low-cost PGRS has been verified and realize feasibility, highly average precision, and good real-time performance of gait recognition. Furthermore, the experimental results reveal the potential for the computer supervision of non-pathological and pathological gaits in the plantar-pressure patterns of children and for providing feedback in the application of gait-abnormality rectification.

In this study, the implementation of the IGRM is mainly done via a GUI program on a computer. Thus, a PC is still the indispensable device used in pathological gait recognition. In the future, all of the PGRS components should be integrated into an embedded, wearable system to constitute a more powerful, practical PGRS. Besides, more subjects must be involved, and more plantar-pressure data should be collected to build a pathological dataset for more complex pathological gait research.

## Data Availability

The datasets used and/or analyzed during the current study are available from the corresponding author on reasonable request.
